# Multiple Large Perineural (Tarlov) Cysts in the Sacrum of a Cadaver: A Case Report and Review

**DOI:** 10.7759/cureus.1156

**Published:** 2017-04-12

**Authors:** Jocelyn Gonzales, Joe Iwanaga, Nitsa Topale, Rod J Oskouian, R. Shane Tubbs

**Affiliations:** 1 Neurosurgery, Seattle Science Foundation; 2 Seattle Science Foundation; 3 Research Division, Seattle Science Foundation; 4 Neurosurgery, Complex Spine, Swedish Neuroscience Institute

**Keywords:** spine, csf, sacral, intrasacral, pain, imaging

## Abstract

Tarlov or perineural cysts are cerebrospinal fluid (CSF)-filled sacs found between the perineurium and epineurium of the nerve roots. It is still unsure whether the origin of these cysts is intradural or extradural. They can either be asymptomatic or create a variety of negative impacts on comfort and quality of life. In this case report, we describe the presentation of multiple Tarlov cysts including one large cyst discovered during a routine cadaveric spinal dissection and the relevant and related literature. To our knowledge, this is the only cadaveric case report of Tarlov cysts and offers an interesting window into their anatomy.

## Introduction

Perineural cysts, commonly referred to as Tarlov cysts, are cerebrospinal fluid (CSF)-filled sacs in between the perineurium and epineurium of the nerve roots [1-2]. They are most common in the sacrum and generally occur at or near the junction of the posterior root and dorsal root ganglion and are bordered by reticular or nerve fibers [2-3]. Clinically, these can compress neighboring nerve fibers and cause neurological symptoms [2]. Most cases of Tarlov cysts are asymptomatic and do not freely communicate with the subarachnoid space [4]. Although many clinical studies have examined the surgical treatments, the pathogenesis and best treatment has yet to be determined and is controversial [2, 5-6]. To our knowledge, there have been no cadaveric studies of the perineural sacral cyst. In this case report, we describe a case of Tarlov cysts found in a cadaveric specimen.

## Case presentation

**Figure 1:**
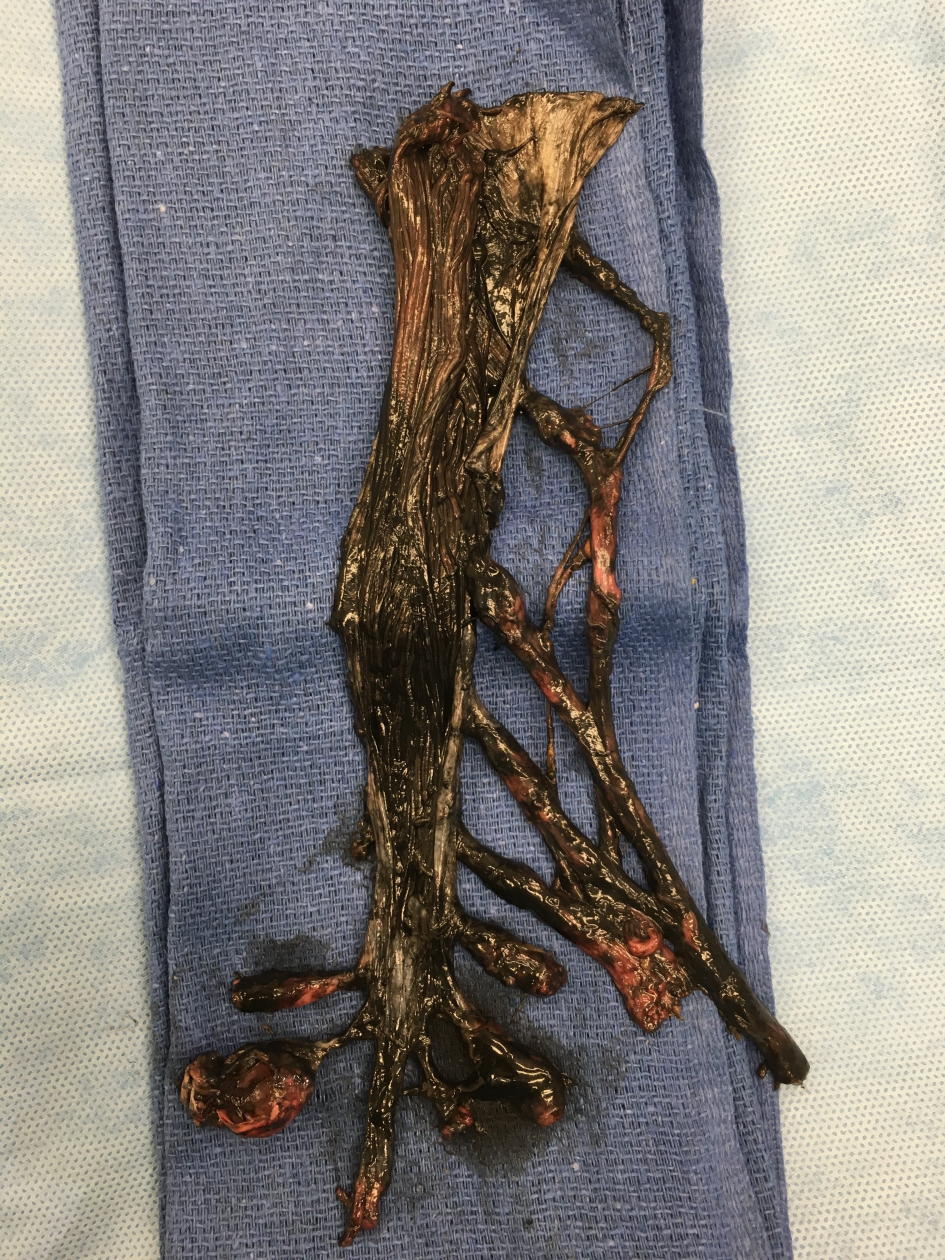
Tarlov cysts dissection.

**Figure 2:**
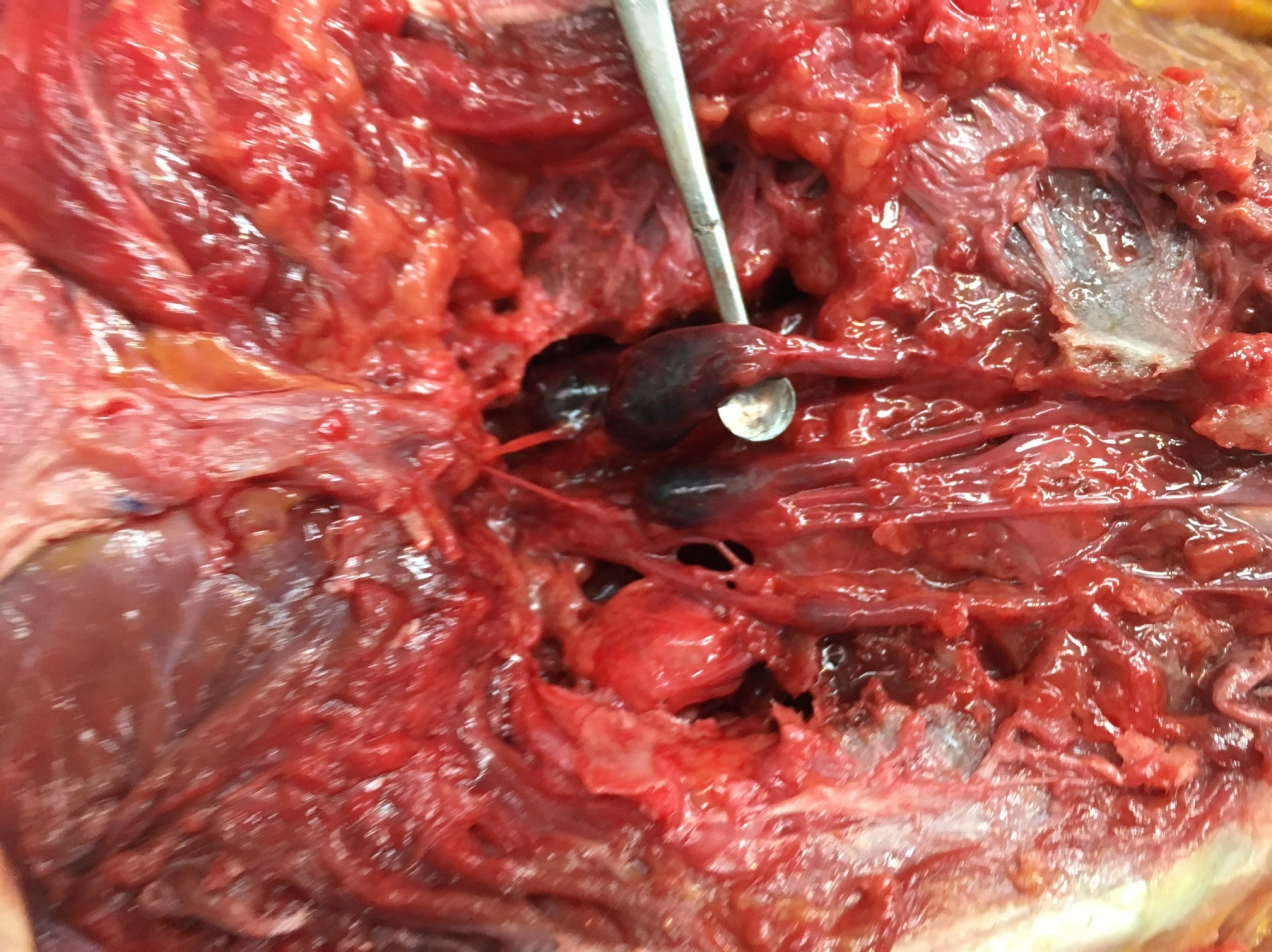
Posterior view of the sacral region with bony elements dissected away to expose the Tarlov cysts.

**Figure 3:**
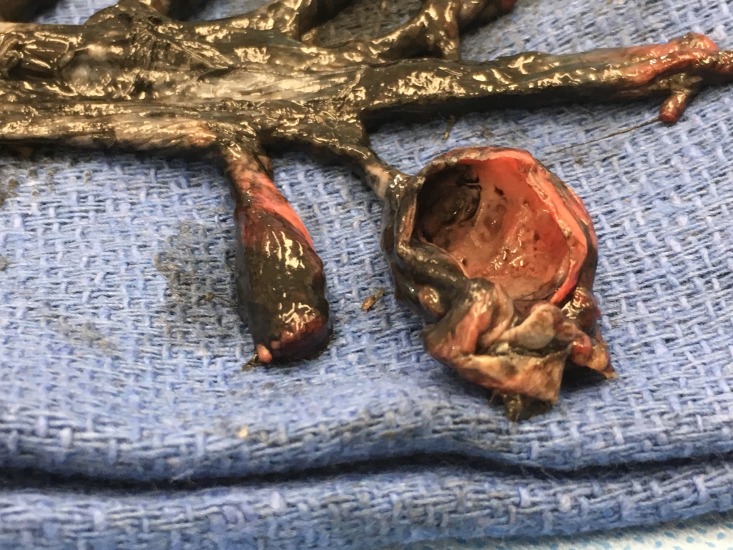
The largest cyst with the anterior surface cut away to show the interior.

During the routine dissection of the spine of a female cadaver (aged 72-years at death) the bone over the dorsal sacrum was found to be extremely thin and almost transparent. With a dissection probe, the posteriorly thinned bone was easily removed. This revealed multiple large intrasacral nerve cysts. In total, there were five cysts found in the sacral region (Figure 1). The largest of these was measured to be 3 × 2.5 cm. Black ink was injected into the subarachnoid space via a cervical spine laminectomy performed at C1 and C2. The posterior elements of the sacrum were removed to expose the intrasacral course of the sacral nerves (Figure 2). The entire distal thecal sac and attached dural nerve root sleeves with associated Tarlov cysts were removed for examination. The surface of the largest cyst was incised and small nerve fibers were detected inside the cyst wall (Figure 3). The injected ink did not flow into the cysts themselves. As a cadaveric examination, the present study did not require approval by an ethics committee at our institution, and the work was performed in accordance with the requirements of the Declaration of Helsinki (64th WMA General Assembly, Fortaleza, Brazil, October 2013).

## Discussion

This represents the only documented report of a cadaveric dissection of the Tarlov cyst in the extant literature, barring the discovery of such cysts by Tarlov during his dissection of 30 cadavers at the Montreal Neurological Institute [4]. Clinical studies have found Tarlov cysts to be present in up to five percent of back pain cases, with less than one percent of those cases being symptomatic due to the cyst [4].

Tarlov cysts have been theorized to be congenital or arise secondarily due to inflammation, trauma, or degenerative processes [2]. Trauma suggests that hemorrhaging impairs the venous drainage between the perineurium and epineurium, causing the build-up of fluid that eventually fills the cyst [2]. On the other hand, a congenital origin suggests that during arachnoid proliferation within the nerve root sleeve, an obstruction to CSF flow forms [7]. In the study by Marino, et al., examination of family histories suggested that Tarlov cysts have both vertical and lateral inheritance, in some cases. A report connecting Tarlov cysts with Marfan syndrome supports a genetic hypothesis for some patients [8]. Hydrostatic pressure might also be a contributing factor and potentially the reason the S2 nerve root is so commonly involved [9].

Symptoms of Tarlov cysts include sacral and ischial pain, urogenital and bowel incontinence, sciatica, coccydynia, and cauda equina syndrome [3, 5, 7]. Symptoms are aggravated by standing, walking, or climbing stairs and are only relieved by recumbence [5-6].

Tarlov cysts are commonly diagnosed on magnetic resonance imaging (MRI). Historically, myelograms demonstrated the lack of early entry of water-soluble contrast into the cyst and therefore aided in the diagnosis [2, 7, 9]. Treatments for Tarlov cysts range from fibrin glue injection, microsurgery, pain management, and conservative treatments such as aspiration or injection of steroids [6-7]. Aspiration of cyst contents is typically temporary with refilling usually in a short period of time [9]. However, if aspiration results in symptom relief then surgical removal of the cyst is more likely to be successful. Tarlov himself popularized the use of fibrin glue injection in nerve repair and found that cyst excision resulted in improvement of symptoms in some patients [4]. Matsumoto, et al. [6] divided Tarlov cyst surgical treatments into three categories: procedures to lower CSF hydrostatic and pulsatile pressures, procedures to decompress cysts, and procedures to shrink cysts. There is no consensus on which surgical procedure is the safest or effective [6]. However, surgical treatments, in general, are discouraged unless severe neurological symptoms are present [7]. Additionally, as the cysts are lined with neural elements, surgery can result in new neurological problems.

## Conclusions

In this case report, we described the occurrence of multiple Tarlov cysts in a cadaveric specimen dissection. The origin of the cyst is still controversial, however, as Tarlov mentioned in his original report, it is difficult to clarify their origin as extradural or intradural.
